# Danger Signals in Anesthesia1Read at meeting: of Canadian Medical Association, Montreal, 1907.

**Published:** 1907-11

**Authors:** Samuel Johnston

**Affiliations:** Toronto


### SELECTED ARTICLES.

### Danger Signals in Anesthesia.[1]

By SAMUEL JOHNSTON, M. A., M. D., Toronto.

[*The Canadian Practitioner, October, 1907.*]

THE subject I have chosen for this paper was taken on account of the general lack of knowledge on the part of the practitioner in the recognition of signs of danger in the administration of anesthetics, or even, when recognised as something unfamiliar in the routine signs of anesthesia, the inability to interpret them.

It is not to be expected that the busy practitioner can have the time or opportunity to make himself familiar with all or even many of them; nevertheless, since he is called to give not a few anesthetics during the year's routine of duties, a few hints on the more commonly met with signs of danger may not come amiss.

Next to the proper method of administration, the recognition of any variation from the usual signs of surgical anesthesia is of the utmost importance, for if one can train himself to recognise the initial approach of danger the real dangers may often be avoided, and no doubt some lives saved. Signs of danger are so varied and so subtle that unless one is giving more time and attention to their recognition than is possible for a general practitioner to do the dangers are realties to be coped with rather than avoided.

It is difficult to point out, simply by means of reading a paper, all the different indications that present themselves to an experienced anesthetist during an administration. He has come to his knowledge through the slow, varied and time-honored process of personal experience, and besides, there do arise some unaccountable conditions of which, from experience, he has learned what the result will be if the warning they convey is not heeded.

It would be a simple matter for the expert to impart his knowledge were he able to demonstrate clinically, in one or two patients, the signs as they appear to him when giving an anesthetic, but in order to do this it would mean possibly as many different

---

1. Read at meeting of Canadian Medical Association, Montreal, 1907.

patients and methods of administration as there are dangers to be met.

Dangers do come unexpectedly even when the most careful administration is given and the closest watch kept; nevertheless, many could be avoided if one studies the signals heralding their approach and is prepared to act promptly and efficiently in averting them.

I will endeavor as briefly as possible to indicate a few of the more frequent signs of danger and the methods of treatment which have been most successful.

Before commencing an administration the anesthetist should acquaint himself with regard to the condition of the patient, and although an elaborate examination is not usually desirable, for he will be told of any preëxisting disorder, still on two important points he must be fully informed, namely, the way in which respiration is performed and the condition of the circulation. He should acquire the habit of gaining an impression of his patient's physical make-up, as to the ease or difficulty, rapidity, depth or shallowness of his breathing, and as to the vigor or feebleness of his circulation. He should note the way the patient moves, speaks and breathes, and carefully observe his color, feel the pulse, and, if there is a suspicion of abnormality within the chest, use the stethoscope. People differ much in their behavior towards anesthetics, and this depends both upon their physical and their psychical natures.

A perfectly healthy individual of fine physical development is very often a difficult subject to anesthetise, for the reason that the greater the muscular development the more prone are mechanical difficulties to arise. Muscular spasm of a firmly-set jaw in an athletic young man is no easy proposition to handle.

The red-faced, short-necked, plethoric or alcoholic individual is very liable during the induction period to have much congestion of the tongue and fauces and spasm of the jaw. It is wise in all such cases, especially when ether is the anesthetic, to insert a small prop, with a string attached, between the teeth.

In the edentulous, if a prop is not inserted or a pledget of gauze placed between the gums and cheeks, obstruction to breathing arises by sucking in the cheeks.

When there is obvious and extensive nasal obstruction, the mouth must be propped so that the patient may receive enough of the anesthetic, with proper admixture of air, to produce smooth anesthesia. In partial obstruction I use small rectal tubes, cut six or seven inches long, passed through the nares to nasopharynx. These, well vaselined, should be placed in position after the patient has been partially anesthetised, and joined with a safety-pin in front, so that they will not slip beyond reach. This is a most excellent method with stout patients, whose tongues swell or fall back.

Pale, weakly individuals are easier to anesthetise than the athletic type and, generally speaking, women more easily than men. Women, are, however, more liable to emotional excitement, but seem to be particularly free from a tendency to muscular spasm.

Very fat persons do not take ether well on account of the congestion of the tongue and fauces and hypersecretion of mucus it produces. With these patients the $C_1E_2$ mixture acts well.

Neurotic or hysterical people are liable to spasmodic muscular contraction, and complete muscular relaxation is more difficult to keep, as a rule, with them than with other patients.

With painful lesions, especially in connection with those of the genitourinary organs, reflex excitability is prone to occur, but certainly the operation should not be begun until this excitable condition is overcome.

For example, in stricture cases, in passing instruments, often when the instrument reaches a particular part reflex spasmodic inspiration is set up, even though there is deep anesthesia. This variation in the breathing may occur when the sphincters are dilated or uterus pulled down.

Very nervous, timid people should be anesthetised with ether rather than chloroform. Even without anesthetics such people have died of fright, and so, owing to the depressing effect of chloroform, ether has the element of safety lacking in the other drug.

Dangerous conditions show themselves in the following ways:

1. Obstruction to respiration, due to foreign bodies such as blood, mucus, loose teeth, etc., congestion of the tongue, fauces, etc., spasm of muscles of the jaw and neck, collapsing cheeks in the edentulous, laryngeal spasm and general respiratory spasm.

2. Depression or failure of respiration.

3. Depression or failure of circulation.

These last two effects may be due to the toxic action of the drug, reflex effect of the operation, or the physical condition of the patient. Depression and even failure of circulation may arise from vomiting, but more frequently in any case with chloroform than with ether as the anesthetic. Therefore the probability of trouble arising, as shown by numerous signals, both in the patient before anesthesia has begun and also after the induction, can to a certain extent be foretold and preventive measures adopted.

When *spasm* and *congestion* occur they are to be met first by pushing forward the jaw behind the angles. If this is not enough, the mouth is to be opened with a gag and the tongue drawn forward with tongue forceps. If the anesthetic is being given with a free supply of air, the spasm will soon pass away. But it may be necessary in thick-necked individuals during anesthesia, and in the edentulous, that the mouth be kept open, the lower jaw pushed well forward and the tongue forceps used from time to time, or the tubes, mentioned before, inserted.

With *foreign bodies*, turn the head to one side and sponge out the offending materials or remove with the finger. When a hard substance has been inhaled the patient must be inverted if passing the finger cannot recover it.

For *laryngeal spasm*, indicated by a high-pitched "crowing" noise with inspiration, occurring usually under chloroform, the treatment is rhythmic tongue traction with the forceps.

*Commencing failure of respiration* is perhaps the most common condition arising during anesthesia, due partly or wholly to the anesthetic. Pallor or blueness of the face, feebleness of breathing, combined with an insensitive cornea, herald this danger. If the administrator stops the anesthetic, rubs the face and lips briskly with a towel, and lowers the head, he will usually succeed in restoring breathing; but if not, the case must be treated as one of respiratory failure. In these latter cases the pupil usually dilates, but it is not a safe sign, for it often remains contracted, especially in infants, until death is imminent.

This incipient respiratory failure must also be distinguished from the very quiet breathing of too light anesthesia. This, however, is easy, as depression in respiration, associated with slow, feeble and irregular pulse, pale or livid countenance, and insensitive cornea, as well as consideration of the length of time of the anesthesia and the previous state of the patient's health, will all have impressed themselves, so that the anesthetist will be alert to impending danger.

In *arrest of breathing* the treatment will be the same whatever the anesthetic employed. Place the patient on his back, with head in line with the body, not above it, and turned to one side. Half open the mouth and draw out the tongue with the tongue forceps; pass the finger to the back of the throat and hook the base of the tongue and epiglottis forward and quickly sponge out the throat. Then firmly compress the chest, placing a hand at each side of the sternum, and using the weight of the body. If breathing does not start now, have an assistant keep the tongue forward and the mouth open, compress the chest again, and if this is not sufficient to reëstablish breathing, seize the patient's arms and do artificial respiration by Sylvester's method. Artificial respiration, by Sylvester's method, and squeezing the chest (Howard's method) are to be repeated slowly, a pause after each compression. Then repeat the series, first inflate, then compress and pause, doing this about fifteen times a minute until breathing begins. Usually a deep sigh signals this return. Be sure and expel the air before beginning to inflate, for the atmosphere in the lungs is laden probably with the anesthetic, and do not carry out the movements too rapidly. This is the essential mode of procedure and of the first importance to be carried out in detail.

Artificial respiration is the great restorer of both cardiac and respiratory action, and it can unaided restore the patient. Time must not be lost getting drugs, injections, etc., for these may fail and valuable time is passing in which artificial respiration would probably have succeeded. However, if further help is available, accessory aids may be used if thought necessary, such as giving a hypodermic injection of strychnia (1-20 to 1-12 of a grain) into the thigh or lower abdomen, rectal injections of one pint of water at 105 deg. F., with two ounces of brandy, brisk friction of the face and gums with a dry towel and bandaging and raising the lower limbs.

If circulatory failure has occurred, compression of the heart between the hands, one on the chest and the other pushing up below the left costal arch, is excellent. The reflex effect of the operation on respiration is usually seen by a deepening and quickening of the breathing when the skin is being incised, but if the patient is not sufficiently anesthetised the beginning of the operation may cause a spasmodic holding of the breath, which in the case of chloroform is very dangerous. However, it should be borne in mind that operations on the rectum and vagina, even when the patient is fully under the influence of the anesthetic, generally cause a noisy, jerky respiration, so much resembling stertor that it is sometimes mistaken for it. Such respiration does not indicate an overdose of the drug.

The *position in which the patient is placed* may interfere so with respiration that it will lead to depression and stoppage of breathing. The most formidable accident in surgical anesthesia is *circulatory failure,* which more often accompanies chloroform anesthesia than any other anesthetic. When an overdose of this drug is given, the quiet breathing of deep anesthesia sometimes becomes noisy and stertorous, the pulse loses its strength, becoming intermittent, flickering and disappearing in a few moments of time. Then breathing grows more and more shallow until both respiration and radial pulse are gone. These are the usual signs. At this point artificial respiration will often restore the breathing and rescue the patient from death. But, on the other hand, the pulse may be beating well, the breathing quiet, the heart and respiration suddenly stop without warning, or one of such an evanescent kind that only the keenest observation will detect it. The fleeting danger-signal alluded to is indicated by a pallor around the mouth projecting upwards beside the alœ of the nose, and if the finger is on the facial or temporal pulse an irregularity and then intermittency occurs. If one is quick enough artificial respiration may be started before the pulse disappears.

Heretofore I have said little about the pupil as a signal when danger is imminent, for I have found that in the case of most anesthetics it is unreliable. In chloroform anesthesia it may, perhaps, be more of a guide, in conjunction with other signs. However, when a patient's pupil is found to be a reliable guide, it affords the earliest signals of danger and the surest signs of safety. The pupil is much contracted, the patient insensible, when no danger is near, but on the slightest amount of over-dosage being given it dilates. Now is the time to resort to measures to effect restoration.

This dilatation of the pupil from overdosage must be distinguished from the same condition when the patient is emerging from the narcosis, and also just before nausea and vomiting ensues. Here the utmost caution is needed. In the latter the patient will show signs of returning consciousness, in the former the condition of deep anesthesia will persist, the pulse will be almost imperceptible and respiration hampered. When the dilatation of the pupil results from returning consciousness, the treatment is a fresh supply of chloroform, which will also usually prevent vomiting and cause the pupils to return to their normal size. In dilatation of the pupil produced by the surgeon's manipulation of sensitive parts, the anesthetist will continue to give the anesthetic.

When *vomiting* occurs in spite of all efforts to prevent it, the head must be turned aside and free outlet given to the vomited matter, and if necessary cleanse the mouth with the finger covered with gauze. The danger to be avoided in this emergency is the sucking backward into the larynx of vomited matter when the patient draws in the deep breath following the emesis.

A word in conclusion. Signs of danger and collapse may arise at any time from the induction period until the last drop of the anesthetic is given. It therefore behooves the administrator to be impressed with the fact that during an administration of any anesthetic eternal vigilance is the price of safety.

---

Syphilis and Massage.—Robert W. Taylor, New York, describes a case of syphilis occurring while the patient was undergoing massage treatment from an operator who had mucous patches in his mouth, and who slobbered and wiped the saliva away from his mouth with his hands. Some benign papules became inoculated by this saliva and produced initial lesions which were followed by a typical attack of syphilis.—*Medical Record,* October 12, 1907.

